# Competing institutional logics in surgical training: a qualitative study of coping strategies in China's dual-track system

**DOI:** 10.3389/fmed.2026.1903226

**Published:** 2026-09-23

**Authors:** Tong Su, Feiyu Zhao, Wenjuan Mo, Zhiting Wang, Yuanyuan Yan, Lei Zhao

**Affiliations:** 1 School of Nursing, University of South China, Hengyang, China; 2 Hunan University of Medicine General Hospital, Huaihua, China; 3 Hengyang Medical School, University of South China, Hengyang, China; 4 Department of Gastrointestinal Surgery, The First Affiliated Hospital of University of South China, Hengyang, China

**Keywords:** clinical competence, institutional logics, medical education, qualitative research, surgical training

## Abstract

**Background:**

In China, clinical medicine professional master's education is closely integrated with standardized residency training, combining academic education with extensive clinical practice. However, trainees may experience competing demands between research-related responsibilities and clinical skill development.

**Methods:**

This qualitative study used a phenomenological design guided by institutional logics theory. Fourteen surgical postgraduates (12 males, 2 females) from a large teaching hospital in southern China were purposively sampled and participated in semi-structured interviews. Data were analyzed using Colaizzi's 7-step method, with rigor ensured through Lincoln and Guba's criteria.

**Results:**

Three interconnected themes emerged. First, institutional anxiety: surgical competence was central to professional identity, yet research tasks displaced training opportunities, generating ontological insecurity. Second, institutional barriers: participants perceived temporal constraints from research demands, an evaluative emphasis on research achievements, and relational barriers related to supervisors’ control over learning opportunities. Third, coping strategies: students developed temporal reappropriation, informal learning communities, strategic visibility, and technology as a learning prosthesis, effectively “practicing in the cracks”. A proposed Institutionally Mediated Model illustrates how macro-level logics shape meso-level mechanisms and micro-level agency.

**Conclusion:**

The findings suggest that, within the integrated professional master's and standardized residency training context, competing research and clinical demands may create temporal, evaluative, and relational barriers to surgical learning. Participants developed individual and collective coping strategies to preserve opportunities for clinical skill development, revealing a potential gap between formal training objectives and experienced learning priorities. System-level efforts are needed to align evaluation practices, protect opportunities for deliberate practice, support supervisors’ educational roles, and integrate effective informal learning resources into surgical training.

## 1 Introduction

Surgical competence is essential for patient safety and professional identity (1). Globally, competency-based medical education has advanced through tools such as the Accreditation Council for Graduate Medical Education (ACGME) Milestones for early identification of underperforming trainees (2), Entrustable Professional Activities (EPAs) for assessing practice readiness (3), and Delphi-derived simulation checklists (4). Structured video grading (e.g., Objective Structured Assessment of Technical Skills (OSATS)) can predict postoperative complications and define learning curves (5). However, systematic reviews indicate that many robotic and urology curricula lack comprehensive validation (6, 7), underscoring the need for evidence-based, multimodal assessment throughout training.

In China, clinical medicine professional master's education is closely integrated with standardized residency training. This broader integration of medical education and clinical training also reflects ongoing reforms in China's medical education system (44). Under the national standardized residency training framework, full-time clinical medicine professional master's students who undertake clinical competency training according to residency requirements are expected to complete at least 33 months of clinical practice, while standardized residency training generally lasts 36 months (8). Clinical training is organized through rotations across relevant departments and follows staged progression, combining clinical practice, theoretical learning, and educational activities. The national training standards emphasize standardized clinical diagnostic and treatment competence and organize training around six core competencies, with specialty-specific knowledge and skills integrated throughout clinical practice (8, 9).

Importantly, this integrated training structure does not mean that clinical and academic requirements are formally equivalent or always experienced as mutually supportive. The national framework also incorporates evidence-based medicine, clinical teaching, and clinical research training, while maintaining clinical practice as the central focus of residency training (10). Thus, postgraduate trainees in academic medical centers may need to negotiate multiple expectations, including clinical competence, academic progression, research productivity, and professional development (11–13, 40–43). These potentially competing demands provide an important institutional context for examining how trainees allocate limited time and navigate tensions between research and clinical learning (14).

The clinical consequences are substantial. Novice surgeons performing complex procedures have higher complication and mortality rates (15). Insufficient operative exposure may have important implications for the development of surgical competence and readiness for independent practice. Moreover,objective assessment approaches such as EPAs and structured video assessment may not yet be consistently implemented across training settings, which may limit opportunities for standardized feedback and remediation (4, 6). Despite documented structural challenges and limited operative exposure (16, 17), few studies have explored how students perceive and navigate these contradictions (18). Understanding their lived experiences is essential to identify hidden mechanisms shaping competence development. Although recent medicine–education cooperation policies have sought to strengthen coordination between medical education and clinical training, challenges in aligning institutional expectations may remain (19). These challenges provide an important context for understanding how trainees navigate competing demands in everyday training. To analyze this, we draw on institutional logics theory, which posits that behavior is shaped by competing institutional logics (20, 21, 38, 39). In Chinese surgical education, “research logic” conflicts with “clinical logic.” These logics manifest in daily practices such as curriculum, assessment, and supervision (22). Students at the intersection must develop coping strategies, often incurring emotional and professional costs. Although this theory has been applied in health professions education (23, 24), it has rarely been used to examine surgical competence development in non-Western settings; existing Chinese studies focus on curriculum or simulation, not micro-level negotiation of tensions (22).

This qualitative study addresses three questions: (1) How do surgical postgraduates perceive surgical competence within the dual-track system? (2) What institutional barriers do they encounter when navigating the tension between research and clinical demands? (3) What coping strategies do they develop, and what hidden curriculum do these strategies reveal? By answering these questions, this study provides empirical evidence from trainees’ perspectives, extends institutional logics theory, and informs policy redesign aimed at achieving balanced surgical training.

## 2 Method

### 2.1 Theoretical framework: institutional logics perspective

This study is guided by institutional logics theory, which posits that individuals’ perceptions, decisions, and actions are shaped by dominant and often competing logics embedded in their institutional environment (19, 20). This theory is particularly suited to analyzing tensions in Chinese surgical education, where residency training lies at the intersection of academic and clinical demands. Unlike theories focused on individual role stress, institutional logics theory foregrounds how structurally embedded contradictions, for example between research productivity and clinical excellence, shape trainees’ strategies for competence development.

In Chinese surgical education, we conceptualize the training environment as an institutional field characterized by two competing logics: research logic (emphasizing publications, grants, and academic reputation) and clinical logic (prioritizing surgical skills, patient care, and clinical reasoning). These logics are not abstract cognitive frameworks but are enacted through daily practices including curriculum design, assessment criteria, supervisor expectations, and resource allocation. The tension between them gives rise to our central theoretical premise: surgical postgraduates’ strategies for developing clinical competence represent adaptive responses to navigating these institutional contradictions.

This framework guided all research phases. (1) Sampling: Participants were purposively selected from those positioned at the intersection of the competing logics, such as residents in academic medical centers where both research output and surgical volume are formally evaluated. (2) Data collection: The semi-structured interview protocol was designed to elicit narratives about how participants experience and navigate institutional tensions, with probes specifically addressing situations in which research and clinical demands conflict. (3) Data analysis: The two logics served as sensitizing concepts, that is, analytic lenses to identify how institutional contradictions manifest in participants’ accounts and shape their reported strategies for surgical competence development. This approach enabled tracing the micro-level consequences of macro-level institutional arrangements.

### 2.2 Setting

This study was conducted from October to December 2022 at a large public teaching hospital in southern China, purposively selected because it provides clinical training forprofessional master's students within the integrated professional master's and standardized residency training framework and has a high volume of surgical postgraduate trainees. Interviews were held in a private office within the gastrointestinal surgery department to ensure confidentiality. Using purposive sampling, 14 full-time clinical medicine professional master's students undergoing surgical training within the integrated professional master's and standardized residency training framework, each having completed at least 14 months of clinical rotations (12 males, 2 females). Sample size was determined by thematic saturation, defined as the point where subsequent interviews yielded no new codes or themes relevant to the research questions (25).

#### 2.2.1 Interview protocol development

The semi-structured interview guide was developed based on a review of the relevant literature and the study's research questions, with Institutional Logics Theory serving as a sensitizing framework. The initial interview guide was pilot tested with one senior surgical resident who had experience with the dual-track training context (The pilot participant was not included in the final study sample). The pilot interview was used to assess the clarity, sequence, and relevance of the questions and probes. Based on the pilot interview, the guide was revised to elicit more detailed accounts of participants’ professional identity, institutional tensions, and coping practices. Specifically, in Theme 1, a probe concerning participants’ desired professional identity and a specific experience illustrating this aspiration was added. In Theme 2, additional probes were introduced to explore how research–clinical conflict affected participants’ daily schedules and plans for surgical learning, whether the training system provided sufficient time for surgical skill development, and how research requirements affected opportunities to observe and practice surgery. These probes addressed temporal, evaluative, and relational dimensions of institutional tensions. In Theme 3, additional probes were added to explore temporal reappropriation, informal peer learning communities, strategic visibility in the operating room, and the use of digital technologies as learning resources. The final semi-structured interview guide is provided in Appendix 1 (Table A1).

#### 2.2.2 Data collection procedures

All 14 interviews were conducted in Mandarin Chinese, the participants’ primary spoken language. All interviews (mean 34 min, range 30–40 min) were conducted in a private office by Author 1 (female, MSc in Nursing, research assistant), who had completed 40 hours of qualitative interviewing training. Participants were informed that the interviewer was a graduate student with no authority over their academic evaluations. A second researcher (Author 2, male, MSc in Medicine) took field notes. Interviews were audio-recorded and transcribed verbatim in Chinese within 24 hours. The transcripts were returned to participants for member checking; no changes were requested, although two participants provided additional reflections. No repeat interviews were conducted because data saturation was achieved after the initial interviews, and no third parties were present.

#### 2.2.3 Data analysis

Data analysis followed Colaizzi's 7-step phenomenological method, with Institutional Logics Theory used as a sensitizing framework. The research questions guided the broad domains of inquiry, while the interview guide included theoretically informed probes concerning potential temporal, evaluative, and relational manifestations of research–clinical tensions. These domains were therefore used to sensitise the researchers to relevant aspects of participants’ accounts but were not treated as rigid, predetermined coding categories. Two authors independently coded all transcripts in Chinese using NVivo 11 (QSR International). Initial coding remained data-driven, with codes generated from participants’ descriptions of their lived experiences. After each coding round, the two researchers compared codes, discussed similarities and differences, and refined the coding structure iteratively. Through repeated comparison and clustering of related codes, patterns concerning time allocation, assessment practices, and supervisory relationships were progressively consolidated into the temporal, evaluative, and relational dimensions of institutional barriers. Thus, these dimensions were informed by the study's theoretical and interview framework but were refined and substantiated through the empirical data. Disagreements were resolved through discussion, with a third author consulted when consensus could not be reached. A hierarchical coding tree was developed iteratively, and the procedural flowchart integrating Colaizzi's steps with the coding process is presented in Figure 1.

**Figure 1:**
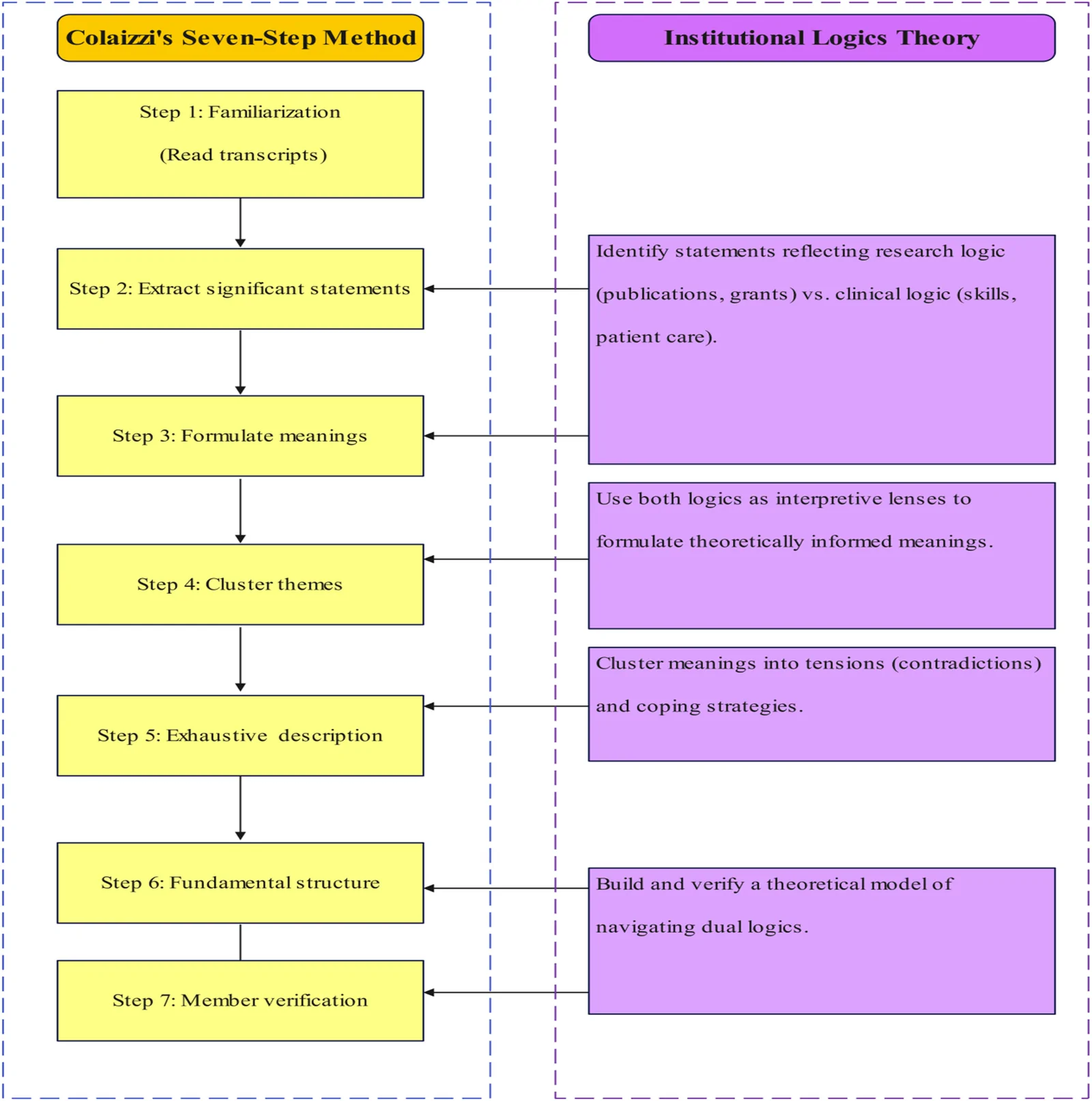
Integration of Colaizzi's seven-step phenomenological method with institutional logics theory.

#### 2.2.4 Translation of quotations

All qualitative analysis was conducted using the original Chinese transcripts. Selected participant quotations were translated into English only after the thematic analysis had been completed and the quotations had been selected for manuscript presentation. The translations were checked against the original Chinese transcripts by members of the research team to preserve the intended meaning, contextual nuance, and participants’ original expressions. Where wording or meaning was ambiguous, the English translation was discussed against the original Chinese text and revised accordingly.

#### 2.2.5 Rigor and trustworthiness

Rigor and trustworthiness were established using Lincoln and Guba's criteria (25). Credibility was ensured through prolonged engagement, member checking, and peer debriefing. Transferability was supported by thick description of the context and participants. Dependability was maintained via an audit trail of sampling, protocol, and coding. Confirmability was strengthened through reflexivity journals and coder triangulation. The interviewer maintained a reflexivity journal to document assumptions (e.g., that research pressure negatively affects surgical training) and discussed these assumptions with a peer debriefer after each interview to mitigate potential bias. She had no prior supervisory or teaching relationship with any participant and informed them that the research would not affect their academic evaluation. Participants were also told that the study was part of the interviewer's master's thesis. After the transcripts and preliminary thematic findings were developed, all 14 participants were invited for member checking. They confirmed that the themes resonated closely with their personal experiences.

#### 2.2.6 Ethical considerations

This study was approved by the Medical Ethics Review Board of a teaching hospital (approval number anonymized). All participants gave written informed consent after receiving detailed information about the study purpose, procedures, and their rights. They were assured that they could withdraw at any time without consequence. To protect confidentiality, audio recordings were deleted after transcription, and transcripts were anonymized. Data are stored on password-protected university servers accessible only to the research team. The study was conducted in accordance with the Declaration of Helsinki.

## 3 Results

### 3.1 Participants

Fourteen surgical postgraduates (12 males, 2 females; age 23–25 years, mean 24.35) participated. All had completed at least 14 months of clinical rotations within the integrated professional master's and standardized residency training framework. Interviews lasted 30–40 minutes (mean 34 minutes). Table 1 presents demographic details.

**Table 1:** Participant characteristics.

ID	Sex	Age	Year of training	Surgical specialty	Completed clinical rotations (months)
P1	Male	24	2nd	Gastrointestinal	12
P2	Male	25	3rd	Hepatobiliary	18
P3	Male	24	2nd	Orthopedics	12
P4	Female	24	2nd	Gastrointestinal	14
P5	Male	25	3rd	Urology	18
P6	Male	23	1st	Cardiothoracic	4
P7	Male	25	3rd	Gastrointestinal	18
P8	Male	24	2nd	Neurosurgery	14
P9	Female	24	2nd	Hepatobiliary	14
P10	Male	25	3rd	Orthopedics	18
P11	Male	24	2nd	Gastrointestinal	14
P12	Male	25	3rd	Urology	18
P13	Male	24	2nd	Cardiothoracic	14
P14	Male	25	3rd	Neurosurgery	18

### 3.2 Overview of thematic structure

Analysis revealed three interconnected themes that form a narrative arc from identity formation to barrier navigation and finally to adaptive agency within the competing institutional logics of surgical education. Guided by institutional logics theory, specifically the tension between research logic (emphasising publications, grants, and academic reputation) and clinical logic (prioritising surgical skills, patient care, and clinical reasoning), the themes are sequentially ordered to reflect how surgical postgraduates experience, interpret, and respond to structural contradictions.

Theme one: “Surgery is the tool for making a living” and addresses professional identity and institutional anxiety. (We use “institutional anxiety” to describe participants’ anxiety arising from perceived constraints and contradictions within their training environment).

Theme two: “Caught between two fires” and addresses institutional barriers.

Theme three: “Practicing in the cracks” and addresses coping strategies.

Collectively, the three themes illustrate a trajectory from logic internalisation (Theme 1) through logic-induced barriers (Theme 2) to adaptive coping under logic conflict (Theme 3), thereby extending institutional logics theory to the micro level of surgical trainees’ lived experiences.

#### 3.2.1 Theme 1 (“surgery is the tool for making a living”) concerns professional identity and institutional anxiety

All 14 participants affirmed surgical competence as central to their professional identity, yet their narratives revealed a duality between vocational calling and anxiety arising from perceived tensions between research demands and clinical training.

##### 3.2.1.1 Subtheme 1.1 concerns surgical competence as the foundation of professional identity

Participants described surgical competence not merely as a skill set but as the essence of being a surgeon.

“Surgical competence is the linchpin of the surgeon's profession. Without it, you’re not a surgeon, you’re just someone with a medical degree.” (P6, male, 1st year)

“The foundation of surgery is the operation itself. Developing surgical competence as a postgraduate is absolutely vital.” (P2, male, 3rd year)

##### 3.2.1.2 Subtheme 1.2 concerns the anxiety of inadequacy, namely “we don’t get enough hands-on experience”

Despite strong identification with surgical competence, participants expressed anxiety about their actual skill levels.

“We’re supposed to be trained as clinicians, but the reality is we spend most of our time on research tasks. The operating room feels like a luxury we can't afford.” (P10, male, 3rd year)

“Sometimes I wonder: if I graduate and go to a hospital, and they expect me to operate, will I be able to? The thought keeps me up at night.” (P13, male, 2nd year)

Students perceived that research tasks displaced surgical training opportunities, generating worry about their readiness for independent practice.

#### 3.2.2 Theme 2 (“caught between two fires”) concerns institutional barriers

Participants identified three mechanisms through which research logic creates barriers to surgical skill development. These mechanisms are temporal, evaluative, and relational.

##### 3.2.2.1 Subtheme 2.1 concerns temporal colonisation, with research tasks occupying most of students’ time

Students reported that research activities occupied the majority of their daily schedule, leaving minimal time for surgical practice.

“A typical day: morning in the OR if I’m lucky, but more often mornings are for writing papers. Evenings and weekends are for more research. When am I supposed to practice surgical skills?” (P3, male, 2nd year)

“My supervisor is under pressure to publish, so he pushes us to focus on research. He says, ’Surgery you’ll learn later, but publications are what get you ahead now.’” (P11, male, 2nd year)

##### 3.2.2.2 Subtheme 2.2 concerns evaluative misalignment, captured by the saying “what gets measured gets done”

“At the end of the day, my graduation depends on my thesis and publication record. Nobody checks how many surgeries I’ve assisted in. So of course I prioritise research; it's what the system rewards.” (P8, male, 2nd year)

“There's a phrase among us: ‘papers are the ticket, surgery is the destination.’ But if you spend all your time getting the ticket, you never actually reach the destination.” (P12, male, 3rd year)

Participants perceived that research outputs were more visible and consequential for postgraduate progression than opportunities to demonstrate surgical competence.

##### 3.2.2.3 Subtheme 2.3 concerns relational dynamics, with supervisors acting as gatekeepers of opportunity

“My supervisor is a brilliant researcher but rarely comes to the OR. When I ask about surgical techniques, he tells me to read a paper instead. I think he sees my value more in terms of helping with his grants than in becoming a skilled surgeon.” (P1, male, 2nd year)

“Some supervisors are great..they really care about your surgical development. But others see you as cheap labour for their research projects. Surgery is an afterthought.” (P4, female, 2nd year)

Students reported marked variability in supervisors’ emphasis on surgical training; some directed students away from the operating room toward research tasks.

#### 3.2.3 Theme 3 (“practicing in the cracks”) concerns coping strategies

Despite the barriers, participants developed strategies to acquire surgical skills outside formal training.

##### 3.2.3.1 Subtheme 3.1 concerns temporal reappropriation, with students finding time in the margins

Students carved out time for deliberate practice by sacrificing sleep, leisure, or social activities.

“I practice knot-tying and suturing in my dorm at night, sometimes until 1 or 2 a.m. It's the only time I have.” (P3, male, 2nd year)

“On weekends, instead of going out with friends, I go to the skills lab. It's usually empty, so I can practice for hours.” (P13, male, 2nd year)

##### 3.2.3.2 Subtheme 3.2: informal learning communities

Students formed peer groups and used digital platforms to share knowledge and practice skills collaboratively.

“Three of us from different surgical specialties meet once a week to practice on each other, not real surgery, but we go through steps, quiz each other on anatomy, and discuss tricky cases.” (P8, male, 2nd year)

“We have a WeChat group where we share surgical videos, interesting cases, and tips.” (P11, male, 2nd year)

##### 3.2.3.3 Subtheme 3.3: concerns strategic visibilityin the operating room

Students actively prepared for cases and volunteered for tasks to build trust with attending surgeons, thereby maximising learning opportunities during limited operating room time.

“When I’m in the OR, I make sure I’m prepared..I study the case beforehand, know the anatomy, anticipate what the surgeon will need. That way, the surgeon trusts me and lets me do more.” (P7, male, 3rd year)

“I always volunteer for the less desirable tasks..retracting, suctioning, whatever. Because if you’re reliable, the surgeon will let you do more.” (P2, male, 3rd year)

##### 3.2.3.4 Subtheme 3.4: concerns the use of technology as a learning prosthesis

Students used online videos, virtual reality simulators, and social media content to supplement limited hands-on experience.

“I watch surgical videos on YouTube and specialised platforms every night. I slow them down, replay critical steps, try to understand why the surgeon does what they do.” (P5, male, 3rd year)

“There are virtual reality (VR) simulators in the hospital, but they’re mostly used for research studies. I managed to get access through a friend and use it whenever it's free.” (P9, female, 2nd year)

### 3.3 Summary of thematic findings

Three interconnected themes emerged. First, participants strongly identified with surgical competence as central to their professional identity but experienced anxiety due to research demands encroaching on training time. Second, institutional barriers operated through three mechanisms: research tasks occupying substantial time, participants’ perceptions of greater salience of research achievements in progression, and variability in supervisors’ support for surgical learning. Third, participants developed coping strategies including temporal reappropriation, informal learning communities, strategic visibility, and technology use. These findings are elaborated with representative quotations in the following sections.

## 4 Discussion

### 4.1 Competing institutional logics and the development of surgical competence

This study explored how surgical postgraduates navigate the tension between research and clinical demands within China's dual-track training system. The findings suggest that surgical competence development is not determined solely by individual motivation or formal curriculum design, but is shaped by the interaction between competing institutional logics, organizational practices, and trainees’ individual agency. Building on institutional logics theory, we propose the Institutionally Mediated Model of Surgical Competence Development (Figure 2), which conceptualizes this process across three interconnected levels: macro-level institutional logics, meso-level organizational mechanisms, and micro-level trainee agency.

**Figure 2:**
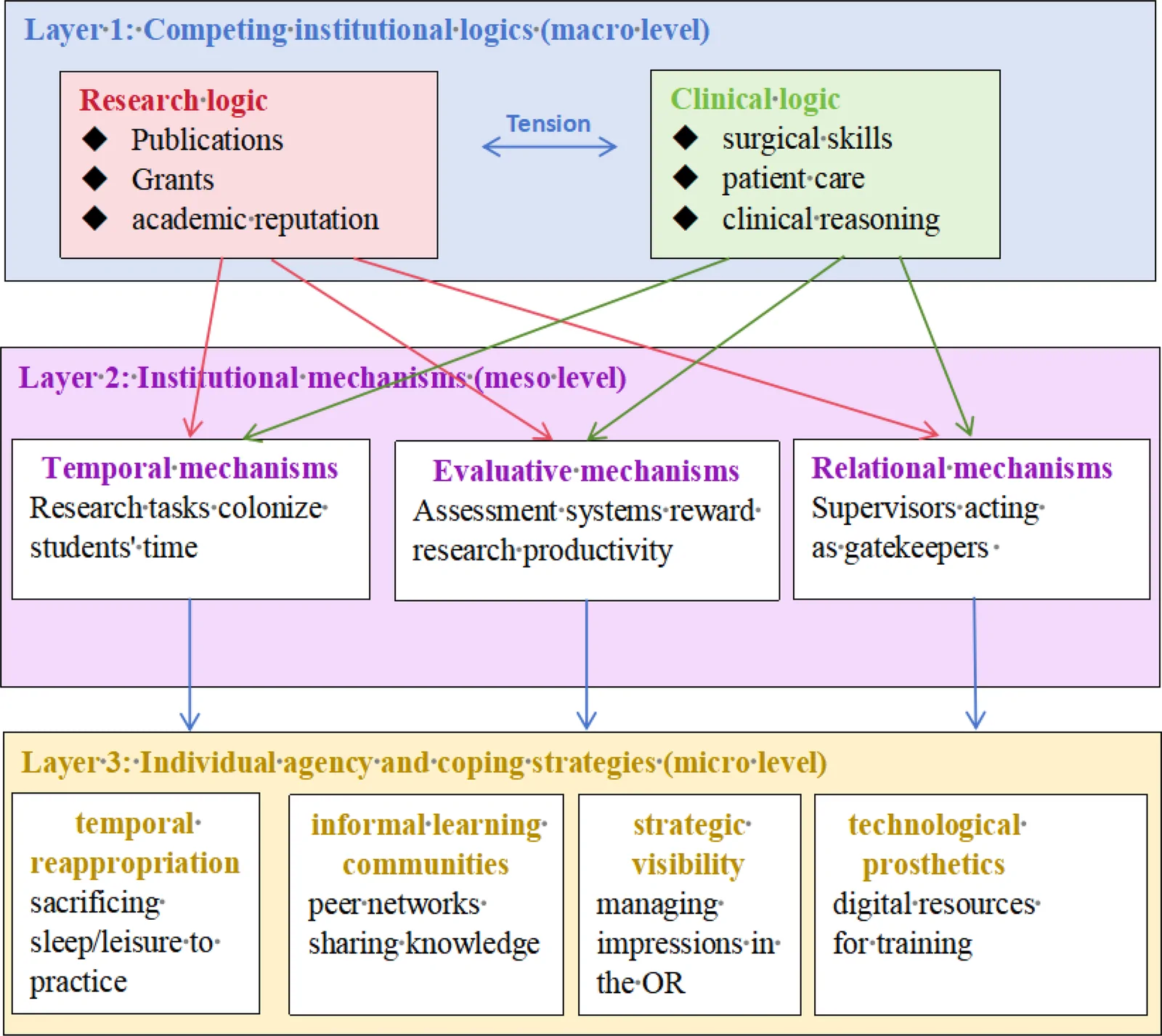
The institutionally-mediated model of surgical competence development.

At the macro level, research and clinical logics represent potentially competing priorities within the training environment. Research logic emphasizes publications, academic productivity, grants, and academic reputation, whereas clinical logic prioritizes surgical skills, patient care, and clinical reasoning. These logics are embedded in institutional policies, evaluation practices, resource allocation, and expectations surrounding postgraduate progression. Rather than operating as abstract principles, they become visible through trainees’ everyday experiences and decisions. This finding extends previous applications of institutional logics theory in medical education by showing how competing institutional priorities can shape the development of clinical competence in surgical postgraduate training.

At the meso level, competing logics are translated into concrete training practices through temporal, evaluative, and relational mechanisms. Research activities can occupy substantial portions of trainees’ schedules, assessment systems can make research productivity more visible and consequential than surgical competence, and supervisors can influence access to clinical learning opportunities. These mechanisms connect institutional priorities with trainees’ everyday learning environments. At the micro level, trainees are not passive recipients of these institutional pressures. Instead, they actively negotiate constraints by reallocating personal time, establishing informal learning communities, increasing their visibility in the operating room, and using digital resources to supplement limited hands-on opportunities.

Above all, these findings suggest that institutional logics mediate not only what trainees are expected to learn, but also when, where, and under what conditions they are able to learn it. This provides an institutionally informed complement to deliberate practice perspectives. Deliberate practice emphasizes repeated, structured, feedback-rich practice as a mechanism of expertise development, whereas our findings indicate that access to such practice is itself institutionally conditioned. When research demands constrain time and supervisory access, opportunities for deliberate practice may become unevenly distributed or displaced by other institutional priorities.

The Institutionally Mediated Model therefore emphasizes a dynamic process rather than a simple top-down relationship. Institutional priorities shape organizational conditions, organizational conditions influence trainees’ learning opportunities, and trainees respond through different forms of agency. This model helps explain how macro-level institutional contradictions become translated into micro-level experiences of surgical training.

### 4.2 Surgical competence, professional identity, and institutional anxiety

Participants consistently described surgical competence as central to their professional identity. This finding is consistent with previous research on surgical identity formation, in which becoming a competent surgeon is closely connected with trainees’ sense of professional purpose and future self. However, our findings add an important dimension: participants’ strong identification with surgical competence coexisted with persistent anxiety about whether they would be adequately prepared for independent clinical practice.

This anxiety appeared to arise not simply from the inherent difficulty of surgical training, but from a perceived mismatch between the kind of doctor participants wanted to become and the opportunities available within their training environment. Participants recognized surgery as the foundation of their professional identity, yet described research activities as occupying time that they believed should be devoted to operative exposure and skills practice. As a result, concerns about insufficient competence extended beyond immediate learning difficulties to uncertainty about their future professional selves.

Drawing on Giddens’ concept of ontological security, this finding can be understood as a form of institutionally produced insecurity. Previous research has documented stress and uncertainty during surgical training (26), but our findings suggest that part of this distress may originate in persistent exposure to competing institutional expectations. Trainees are expected to develop as clinically competent surgeons while simultaneously responding to strong research demands. When these demands compete for the same limited time and resources, trainees may question whether their actual training trajectory is consistent with the professional identity they aspire to achieve.

This distinction shifts the interpretation of trainee anxiety from an individual psychological problem to an institutional and educational issue. Participants’ concerns about whether they would be able to perform surgery after graduation reflect not simply a lack of confidence, but uncertainty about whether the training system is providing adequate opportunities to acquire the competencies expected of them. In this sense, institutional anxiety represents one of the micro-level consequences of competing institutional logics.

The findings therefore suggest that surgical competence should be understood not only as an educational outcome but also as a component of professional identity formation. When institutional arrangements repeatedly constrain opportunities for clinical learning, the consequences may extend beyond skill acquisition to trainees’ confidence in becoming the surgeons they intend to become.

### 4.3 Institutional mechanisms and supervisors as institutional actors

Our findings identified three interconnected mechanisms through which competing research and clinical logics influenced surgical training: temporal, evaluative, and relational mechanisms. These findings extend previous research that has commonly attributed limited operative exposure to logistical factors such as case availability, scheduling, or workload (27, 28). Our findings suggest that limited surgical learning may also result from a deeper institutional misalignment between what is formally rewarded and what is clinically required.

#### 4.3.1 Temporal mechanisms

The first mechanism concerned the allocation of trainees’ time. Participants described research activities as occupying substantial portions of their daily schedules, leaving limited opportunities for deliberate surgical practice. The issue was therefore not simply that trainees had “too much work,” but that the two institutional logics competed for the same finite resource: trainees’ time. Research tasks produced visible and measurable outputs, whereas the benefits of repeated surgical practice were often less immediately visible within the existing evaluation system.

This temporal imbalance may have implications for expertise development. Surgical skills require repeated practice, observation, feedback, and progressive responsibility. When opportunities for these activities are repeatedly displaced by research tasks, trainees may be unable to accumulate sufficient experience through structured clinical learning. The resulting coping strategies, including practicing at night or during weekends, further demonstrate how trainees attempt to recover learning time that has been displaced by institutional demands.

#### 4.3.2 Evaluative mechanisms

The second mechanism concerned assessment. Participants perceived research outputs, particularly publications and thesis-related achievements, as more visible and consequential for their progression than surgical competence. This creates an evaluative asymmetry: research productivity is represented through tangible outputs that can be counted, documented, and compared, whereas surgical competence may be assessed less consistently. This finding should be interpreted as a perception of relative salience rather than an absence of formal clinical competency assessment.

Participants perceived research-related achievements as more immediately consequential for progression, even though clinical competence remains an explicit component of residency training. The participant observation that “papers are the ticket, surgery is the destination” captures this contradiction. The metaphor suggests that research output is perceived as a prerequisite for progression, whereas surgical competence represents the ultimate professional goal. When the former becomes more consequential within formal assessment, trainees may rationally prioritize research even when they recognize the importance of clinical learning.

This finding is consistent with the institutional logics perspective because individuals’ choices are shaped by the incentives embedded in their institutional environment. From the trainees’ perspective, prioritizing research does not necessarily reflect a lack of commitment to surgery. Instead, it may represent a rational response to an evaluation system in which research achievements are more immediately visible and consequential.

#### 4.3.3 Relational mechanisms: supervisors as institutional actors

The third mechanism concerned supervisory relationships. Participants described substantial variability in supervisors’ emphasis on research and clinical training. Some supervisors directed trainees toward research tasks, while others actively protected opportunities for surgical learning. This finding is consistent with previous work highlighting the importance of supervisors in intraoperative teaching (29), but our findings extend this literature by positioning supervisors as institutional actors who themselves navigate competing research and clinical demands.

Supervisors may simultaneously be expected to produce research outputs, maintain clinical responsibilities, and supervise postgraduate students. Consequently, their decisions about allocating trainees’ time and assigning learning opportunities may reflect not only personal teaching preferences but also institutional incentives and constraints. Prioritizing research should therefore not necessarily be interpreted simply as “bad teaching.” Rather, it may represent an adaptive response to institutional career incentives and expectations.

At the same time, the presence of supervisors who protected surgical training opportunities indicates that institutional logics do not determine behaviour mechanically. Two participants specifically described supervisors who actively protected time for clinical learning. These cases suggest that supervisors may negotiate, reinterpret, or partially resist dominant institutional priorities. Thus, supervisors occupy an important meso-level position through which institutional logics are either reinforced or moderated in everyday training.

Our study also highlights the need to avoid conceptualizing supervisors solely as barriers to surgical learning. They may simultaneously function as gatekeepers, negotiators, and potential protective factors. Understanding the conditions under which supervisors are able to protect clinical learning opportunities may therefore be important for future educational interventions.

### 4.4 Trainee agency, coping strategies, and the hidden curriculum

Although the institutional barriers described above, participants demonstrated considerable agency in maintaining their surgical learning. Four interconnected coping strategies were identified: temporal reappropriation, informal learning communities, strategic visibility, and technology as a learning prosthesis. These strategies demonstrate that trainees actively negotiate institutional constraints rather than simply accepting them.

Temporal reappropriation involved reclaiming time for surgical practice by sacrificing sleep, leisure, or social activities. Although this strategy enabled trainees to continue practicing basic skills, it also reveals an important contradiction: participants perceived that their formal training schedules did not consistently provide sufficient protected time for deliberate practice, so trainees created such opportunities through personal sacrifice. This form of agency therefore comes at an individual cost and should not be interpreted as a sustainable substitute for institutional support.

Informal learning communities provided trainees with opportunities to exchange knowledge, discuss cases, practice skills, and share surgical videos. These peer-based networks functioned as alternative learning spaces when formal training opportunities were limited. Their emergence demonstrates that trainees can create educational resources and communities outside formal curricular structures.

Strategic visibility involved deliberately preparing for cases, demonstrating reliability, and volunteering for tasks in the operating room in order to build trust with attending surgeons and increase access to hands-on learning. This strategy illustrates how trainees negotiate relational mechanisms at the micro level. When operative opportunities are limited, trainees learn not only surgical techniques but also how to navigate the social conditions through which learning opportunities are distributed.

Technology as a learning prosthesis involved using online surgical videos, virtual reality simulators, and social media-based instructional resources to supplement limited hands-on experience (46). Technology therefore served as an adaptive response to institutional constraints. However, the findings also highlight an important limitation: self-directed digital learning cannot fully substitute for supervised clinical practice because it may lack expert feedback, standardized assessment, and opportunities for real-time correction.

These strategies reveal a gap between the formal curriculum and the hidden curriculum of surgical training. Formally, surgical competence is recognized as an important training objective. In practice, however, participants learned that research productivity could be more consequential for progression and that surgical skills often had to be acquired through informal, self-directed, or opportunistic means. Students therefore learned not only surgical techniques but also how to “practice in the cracks” of the institutional system.

This finding extends the institutional logics perspective by demonstrating how individual agency operates within institutional constraints. Trainees do not simply resist institutional logics; they selectively negotiate them. Their coping strategies allow them to preserve valued aspects of clinical identity while adapting to an environment in which research demands were perceived to compete strongly with clinical learning priorities. At the same time, the need for such strategies indicates that individual agency cannot fully compensate for structural misalignment.

### 4.5 Implications for surgical education and policy

The findings have several implications for improving surgical training within the integrated professional master’s and standardized residency training framework and for translating medical education research into educational practice (45).

Firstly, evaluation systems should be more closely aligned with clinical competencies. Research productivity and academic achievements should not be the only highly visible indicators of postgraduate success. Competency-based assessment approaches, including Objective Structured Assessment of Technical Skills (OSATS), surgical logbooks, and direct observation (30–32), could provide more explicit recognition of surgical competence. Such approaches may help reduce the evaluative asymmetry between research output and clinical performance.

Secondly, protected time for deliberate surgical practice should be established. Training programs could designate regular, protected periods for supervised skills-laboratory practice, allowing trainees to develop technical skills without competing directly with research tasks. This would shift responsibility for securing practice time from individual trainees to the training system and provide a more sustainable foundation for competence development.

Thirdly, supervisory roles and incentives should be reconsidered. Institutions could recognize clinical teaching and mentorship within promotion and performance evaluation systems and provide supervisors with structured training in intraoperative teaching (33). Such changes may reduce the tension between supervisors’ academic incentives and their educational responsibilities and create conditions in which supervisors can protect trainees’ clinical learning opportunities without compromising their own career progression.

Fourthly, informal learning structures should be recognized and appropriately supported. Peer learning groups, curated surgical video resources, simulation facilities, and other forms of trainee-led learning could be incorporated into the formal curriculum. Rather than forcing trainees to rely on hidden or informal learning opportunities, training programs could transform effective coping strategies into supported educational resources.

Finally, sex-sensitive support should be considered in surgical training. Although only two women participated in this study and the findings cannot support broad conclusions about sex differences, their accounts suggested that the combined demands of surgical practice and research may be experienced differently. Existing literature has documented sex-related barriers in surgical training (34–37). Future programs should therefore consider inclusive mentorship, work–life support, and equitable access to clinical learning opportunities while avoiding assumptions based on sex.

In conclusion, these implications point toward a shift from asking trainees to individually “cope better” with competing demands toward addressing the institutional conditions that generate those demands. The central challenge is not to eliminate research from surgical postgraduate education, but to create a training environment in which research and clinical development are mutually supportive rather than structurally competing priorities.

### 4.6 Limitations and future research

This study has several limitations. The study was conducted at a single institution, which may limit the transferability of the findings to other hospitals, regions, or postgraduate training contexts. Future multicentre qualitative studies could examine whether similar research–clinical tensions occur across different institutional settings and training programs.

Besides, the sample included 12 male and 2 female participants. The small number of female participants limits the extent to which sex-related experiences can be interpreted and compared. Future studies should include more female surgical trainees to explore how sex may intersect with competing institutional logics and access to clinical learning opportunities.

Furthermore, the cross-sectional design captures participants’ experiences at a single point during training. Longitudinal research could examine how perceptions of surgical competence, professional identity, coping strategies, and institutional pressures change as trainees progress through postgraduate education and transition into independent clinical practice.

Despite these limitations, the study provides an institutionally informed account of surgical competence development from trainees’ perspectives. By connecting institutional logics with organizational mechanisms and individual agency, the Institutionally Mediated Model offers a framework for understanding how broader institutional arrangements shape everyday opportunities for surgical learning.

## 5 Conclusion

Guided by institutional logics theory, this qualitative study examined how Chinese surgical postgraduates navigate competence development within the dual-track system. The findings show that competing research and clinical logics are translated into temporal, evaluative, and relational mechanisms that shape trainees’ opportunities for surgical learning. In response, trainees develop individual and collective coping strategies, revealing a potential gap between formal training objectives and participants’ experienced learning priorities. The proposed Institutionally Mediated Model illustrates how macro-level institutional priorities, meso-level training mechanisms, and micro-level trainee agency interact to shape surgical competence development. System-level reforms are therefore needed to align evaluation, protect opportunities for deliberate practice, support supervisors’ educational roles, and integrate effective informal learning resources into formal surgical training.

## Data Availability

The original contributions presented in the study are included in the article/Supplementary Material, further inquiries can be directed to the corresponding authors.
