# Supplementary material for: Competing institutional logics in surgical training: a qualitative study of coping strategies in China's dual-track system
Source: Front Med (Lausanne). 2026 Sep 23;13:1903226. doi: 10.3389/fmed.2026.1903226 (PMC13641997; doi:10.3389/fmed.2026.1903226)
Supplement: Supplementary file 1 [file Supplementaryfile1.docx]

Supplementary Material

**Appendix 1 Interview Protocol Development – Detailed Methods**

1. Overview

The interview protocol was developed following a three-stage process: (1) literature review and theoretical mapping, (2) pilot testing, and (3) finalization of the semi-structured guide. This appendix provides the complete protocol and a procedural flowchart.

2. Stage 1: Literature Review and Theoretical Mapping

We reviewed literature in three domains: surgical education (competency frameworks), institutional logics theory (research logic vs. clinical logic), and Chinese medical education reform (the “dual-track” system). Key concepts (research logic, clinical logic, institutional tension, and coping strategy) were operationally defined and mapped onto potential interview questions.

3. Stage 2: Pilot Testing

A pilot interview was conducted with one senior surgical postgraduate student (Year 3) who met the same inclusion criteria but was not included in the final sample. The pilot assessed:

(a) Question clarity and relevance;
(b) Interview duration (final duration: 30-40 minutes);
(c) Effectiveness of probes.

Feedback led to: (a) rewording of two questions for clarity; (b) addition of three probes (marked with * in the final protocol) to elicit deeper reflections on resource allocation and ethical dilemmas.

4. Stage 3 Final Semi-Structured Interview Protocol

The final guide consists of three sections, aligned with the research questions (RQs). All questions are open-ended; probes are used flexibly.

**Table A1. Final Semi-Structured Interview Protocol**

| Section | Question | Probes |
| --- | --- | --- |
| Perceptions of Surgical Competence | Q1. “When you hear ‘surgical competence’, what does it mean to you personally, and how important is it for your future career?” | “Some say ‘surgery is the tool for making a living’, do you agree, and why?” “What specific skills or attributes do you consider most essential?” |
|  | Q2. “How would you compare the importance of surgical skills versus research publications for your career progression?” | “What messages have you received from supervisors, peers, or hospital policies about this?” |
| Institutional Barriers and Tensions | Q3. “Walk me through a typical day or week: what tasks occupy most of your time, and what helps or hinders your surgical skill development?” | “How much time do you spend on research-related activities (e.g., writing papers, analyzing data) versus clinical/surgical activities?” “Have you ever had to choose between attending a surgery and meeting a research deadline? How did you handle it?” |
|  | Q4. “How do research requirements (e.g., publishing, thesis writing) affect your opportunities to practice surgery?” | “Can you give a specific example of a conflict between research and clinical training?” |
| Coping Strategies and Hidden Curriculum | Q5. “Despite challenges, how do you manage to improve your surgical skills? What strategies have you developed?” | “What support (from supervisors, peers, or hospital) would be most helpful?” “Can you give a specific example of an innovative way you have practiced or learned surgical skills outside formal training?” |
|  | Q6. “If you could change one thing about your training environment to better support surgical competence, what would it be and why?” | “What advice would you give to incoming surgical postgraduates based on your experience?” |

**Supplementary 1:Semi-structured Interview Guide**

**Interview Themes**

1. Perceptions of Surgical Competence
2. Institutional Barriers and Tensions
3. Coping Strategies and Hidden Curriculum

**Revised Semi-Structured Interview Guide**

**Opening Script**

“Hello. Thank you very much for taking the time to participate in this interview. I am a research assistant with a Master of Science in Nursing. I am currently conducting a study on the learning experiences of surgical postgraduates under the 'dual-track' training model.”

**Purpose:** “We would like to understand, from your perspective, how you view the development of surgical competence amidst the current demands of completing clinical rotations while also producing research outputs. Specifically, we want to learn about the concrete difficulties you have encountered and the ways you have found to improve your surgical skills.”

**Confidentiality:** “All interview content will be kept strictly confidential and used only for academic research. In the final report, your personal information will be anonymized. The interview is expected to take 30–40 minutes.”

**Recording permission:** “To accurately record your views, I would like to audio-record our conversation. The recording will also be kept strictly confidential and will be destroyed after transcription. Do I have your consent?”

**Voluntary participation:** “You may skip any question or terminate the interview at any time.”

**Theme 1: Perceptions of Surgical Competence**

1. Personally, what does “surgical competence” mean to you? And how important is it for your future career development?

Probes:

(1) Some people say “surgery is the tool for making a living”, do you agree, and why ?
(2) What specific skills or attributes do you consider the most essential components of surgical competence? (e.g., technical skill, clinical decision-making, perioperative management)
2. How would you compare the importance of surgical skills versus research publications for your current graduation and long-term career progression?

Probes:

(1) What concrete experiences give you this impression? (e.g., feedback from supervisors, departmental expectations, graduation requirements, award criteria)
(2) What kind of doctor you want to become? Can you describe a specific moment?

**Theme 2: Institutional Barriers and Tensions**

1．Walk me through a typical day or week: what tasks occupy most of your time, and what helps or hinders your surgical skill development?

Probes:

(1) Specifically, approximately how much of your time is taken up by research-related activities (e.g., literature review, experiments, writing papers, data analysis) versus clinical/surgical activities?
(2) Have you ever faced a conflict between attending a surgery and meeting a research deadline? How did you handle it, and what were your inner feelings at the time?
2．How do the research requirements for graduation (such as publishing papers, completing your thesis) affect your opportunities to participate in and practice surgery?

Probes:

(1) Can you give a specific example of a conflict between research tasks and clinical training that left a deep impression on you?
(2) How has this conflict changed your daily schedule or your plans for surgical learning?
(3) Do you think the current training system provides enough time for surgical skill development? Why or why not?
3．To what extent do you feel that research requirements (e.g., publishing papers, completing a thesis) impact your opportunities to observe and practice surgery? At what levels does this impact manifest?

Probes:

(1) Temporal: Does research work colonize time that you feel should be spent in the operating room or practicing skills?
(2) Evaluative: Does the current assessment system (e.g., graduation hinging on publications) force you to prioritize research?
(3) Relational: What role does your supervisor play in your surgical skill development? Do they act more as a research project leader or a clinical teacher? How does this affect your opportunities for hands-on surgical practice?

**Theme 3: Coping Strategies and Hidden Curriculum**

1．Despite these research pressures, what methods do you use to continuously improve your surgical skills? What strategies have you developed?

Probes:

(1) What support (from supervisors, peers, or hospital) would be most helpful? Can you give a specific example of an innovative way you have practiced or learned surgical skills outside the formal training curriculum?
(2) Temporal Reappropriation: Have you ever sacrificed sleep, rest, or social time to practice basic surgical skills (e.g., knot-tying, suturing)?
(3) Informal Learning Communities: Have you formed spontaneous study groups with peers, or used platforms like WeChat to share surgical videos and discuss cases?
(4) Strategic Visibility: During rotations or when assisting in surgery, what preparations do you deliberately make (e.g., studying the case beforehand, volunteering for tasks like retracting and suctioning) to maximize your learning opportunities?
(5) Technology as a Prosthesis: Do you use online surgical videos, VR simulators, or instructional content from social media for self-improvement? What role do these resources play?
2．If you could change one thing about the hospital's training system to genuinely support your surgical competence development, what would it be and why?

Probes:

Based on your personal experience, what concrete advice would you give to incoming surgical postgraduates to help them better balance research and clinical work and grow into competent surgeons?

**Closing**

“Those are all the questions I have prepared. Is there anything important about this topic that I haven’t asked but you would like to share?”

“Thank you very much for your sharing. Your experiences and feelings are very valuable for understanding the real learning dynamics under the dual-track system. After we transcribe the conversation, we will organize the key points and check them with you. Thank you again for your time and openness.”

**Interviewer’s Guide**

**Preparation Before the Interview**

Review study objectives thoroughly.

Test recording equipment (audio) and prepare backup batteries.

Prepare printed copies of the consent form and the interview guide (for reference).

Arrange a quiet, private room (office, vacant consultation room, or dedicated research room) with no non-participants present.

Ensure participant code (e.g., P1, P2) is assigned and recorded on a separate master list.

**Interviewer Roles and Conduct**

First author (T.S., F.Y.Z) leads the interview, manages flow, and asks most questions.

Corresponding author (Y.Y.Y.) focuses on clinical aspects and may ask follow-up probes when needed.

Both interviewers should:

Use open-ended questions.

Avoid leading or judgmental comments.

Allow silence for the participant to think.

Take brief notes on non-verbal cues or key points (for reflexivity logs).

**Probes and Follow-up Techniques**

Use neutral prompts: “Could you tell me more about that?”, “What happened next?”, “How did that make you feel?”

If a participant gives a short answer, ask: “Could you give me an example?”

Avoid interrupting; let the participant complete their thought.

**Reflexivity and Bias Management**

Immediately after each interview (within 24 hours), both interviewers independently complete a reflexivity log, documenting:

Emotional reactions.

Any assumptions or biases that emerged.

Key non-verbal observations.

Weekly team meetings are held to discuss reflexivity logs and consensus coding.

**Post-Interview Procedures** ( Tong Su)

Transcribe verbatim within 24 hours .

Anonymize by removing any identifying information (replace names, hospital references with codes).

Send transcript to participant via encrypted email for member checking (accuracy and completeness).

Store data on encrypted devices with restricted access.

**Ethical Considerations Throughout**

Remind participants of their right to withdraw at any time without consequences.

Do not pressure for answers; respect “skip” or “I don’t know” responses.

Ensure the corresponding author (Y.Y.Y., L.Z.) is not present during recruitment or consent for participants with prior clinical relationships to avoid coercion.

**Supplementary 2. Informed Consent**

**Dear Postgraduate Student:**

This study aims to explore the authentic experiences, emotional responses, and coping strategies of surgical postgraduates when navigating the institutional contradictions between research and clinical demands under China’s medical education dual-track system. It seeks to reveal how the competing institutional logics shape surgical competence development, and to provide empirical evidence for the optimization of surgical training policies and curriculum design. Please read this document carefully before signing. If you have any questions, please feel free to contact the research team at any time.

1. **Basic Information**

Name: Gender: Age: Grade of postgraduate study:

Specialty/Department: Duration of completed clinical rotations (months):

2. **Study Purpose and Procedures**

This qualitative study explores how surgical postgraduates experience and navigate competing research and clinical demands. You will participate in one individual, semi‑structured interview (approximately 30-40 minutes) in a private office. The interview will be audio‑recorded, transcribed, anonymized, and the recording destroyed. De‑identified findings may appear in academic publications or presentations.

**3. Data Usage Methods**

3.1 All data will be de-identified to ensure that you cannot be personally identified.

3.2 Data may be presented as excerpts, summaries, tables, or figures in academic publications and presentations.

3.3 Your real name, contact information, address, and any other private details will not be disclosed.

3.4 You have the right to be informed about the final form and venues in which the data will be published.

**4. Your Rights**

4.1 Voluntary participation: Your decision to participate is entirely voluntary and will not affect your academic evaluations, clinical placements, or your relationship with supervisors or the department in any way.

4.2 Right to withdraw: You may withdraw your consent at any time before formal publication of the research findings. If you withdraw, we will immediately cease using your data and destroy all related materials.

4.3 Right to be informed: You may request detailed information about how your data will be used and the publication platforms.

4.4 No risk assumption: Participation will not impose any financial burden or legal liability on you. Should any topic cause you discomfort during the interview, you may skip the question or pause the conversation at any time.

**5. Signature Statement**

I have read and understood the above information. I am aware of the study’s basic purpose, procedures, and my rights, and I voluntarily consent to participate in this qualitative study.

Thank you very much for your support!

Participant signature:

Researcher signature:

Date:

**Supplementary 3. COREQ checklist**

**COREQ (COnsolidated criteria for REporting Qualitative research) Checklist**

A checklist of items that should be included in reports of qualitative research. You must report the page number in your manuscript where you consider each of the items listed in this checklist. If you have not included this information, either revise your manuscript accordingly before submitting or note N/A.

| **Topic** | **Item No.** | **Guide Questions/Description** | **Reported on Page No.** |
| --- | --- | --- | --- |
| **Domain 1: Research team and reﬁexivity** | | | |
| *Personal characteristics* | | | |
| Interviewer/facilitator | 1 | Which author/s conducted the interview or focus group? | 5 |
| Credentials | 2 | What were the researcher’s credentials? E.g. PhD, MD | 5 |
| Occupation | 3 | What was their occupation at the time of the study? | 5 |
| Gender | 4 | Was the researcher male or female? | 5 |
| Experience and training | 5 | What experience or training did the researcher have? | 5 |
| *Relationship with participants* | | | |
| Relationship established | 6 | Was a relationship established prior to study commencement? | 6 |
| Participant knowledge of the interviewer | 7 | What did the participants know about the researcher? e.g. personal goals, reasons for doing the research | 5 |
| Interviewer characteristics | 8 | What characteristics were reported about the inter viewer/facilitator? e.g. Bias, assumptions, reasons and interests in the research topic | 6 |
| **Domain 2: Study design** | | | |
| *Theoretical framework* | | | |
| Methodological orientation and Theory | 9 | What methodological orientation was stated to underpin the study? e.g. grounded theory, discourse analysis, ethnography, phenomenology,content analysis | 4 |
| *Participant selection* | | | |
| Sampling | 10 | How were participants selected? e.g. purposive, convenience, consecutive, snowball | 4 |
| Method of approach | 11 | How were participants approached? e.g. face-to-face, telephone, mail, email | 5 |
| Sample size | 12 | How many participants were in the study? | 4 |
| Non-participation | 13 | How many people refused to participate or dropped out? Reasons? | 5 |
| *Setting* | | | |
| Setting of data collection | 14 | Where was the data collected? e.g. home, clinic, workplace | 4 |
| Presence of non-participants | 15 | Was anyone else present besides the participants and researchers? | 5 |
| Description of sample | 16 | What are the important characteristics of the sample? e.g. demographic data, date | 7 |
| *Data collection* | | | |
| Interview guide | 17 | Were questions, prompts, guides provided by the authors? Was it pilot tested? | 5 |
| Repeat interviews | 18 | Were repeat inter views carried out? If yes, how many? | 5 |
| Audio/visual recording | 19 | Did the research use audio or visual recording to collect the data? | 5 |
| Field notes | 20 | Were ﬁeld notes made during and/or after the inter view or focus group? | 5 |
| Duration | 21 | What was the duration of the inter views or focus group? | 5 |
| Data saturation | 22 | Was data saturation discussed? | 4 |
| Transcripts returned | 23 | Were transcripts returned to participants for comment and/or correction? | 5 |
| **Domain 3: analysis and findings** | | | |
| *Data analysis* | | | |
| Number of data coders | 24 | How many data coders coded the data? | 6 |
| Description of the coding tree | 25 | Did authors provide a description of the coding tree? | 6 |
| Derivation of themes | 26 | Were themes identiﬁed in advance or derived from the data? | 7 |
| Software | 27 | What software, if applicable, was used to manage the data? | 6 |
| Participant checking | 28 | Did participants provide feedback on the ﬁndings? | 6 |
| *Reporting* | | | |
| Quotations presented | 29 | Were participant quotations presented to illustrate the themes/ﬁndings? Was each quotation identiﬁed? e.g. participant number | 8-12 |
| Data and ﬁndings consistent | 30 | Was there consistency between the data presented and the ﬁndings? | 7-18 |
| Clarity of major themes | 31 | Were major themes clearly presented in the ﬁndings? | 7-12 |
| Clarity of minor themes | 32 | Is there a description of diverse cases or discussion of minor themes? | 8-12,17 |

Developed from: Tong A, Sainsbury P, Craig J. Consolidated criteria for reporting qualitative research (COREQ): a 32-item checklist for interviews and focus groups. *International Journal for Quality in Health Care*. 2007. Volume 19, Number 6: pp. 349 – 357

**Once you have completed this checklist, please save a copy and upload it as part of your submission. DO NOT include this checklist as part of the main manuscript document. It must be uploaded as a separate file.**
